# The psychophysics of home plate umpire calls

**DOI:** 10.1038/s41598-024-52402-y

**Published:** 2024-02-01

**Authors:** Kevin S. Flannagan, Brian M. Mills, Robert L. Goldstone

**Affiliations:** 1 Department of Political Science, University of California - San Diego, La Jolla, CA 92093 USA; 2 Department of Kinesiology and Health Education, University of Texas at Austin, Austin, TX 78712 USA; 3 Department of Psychological and Brain Sciences, Indiana University, Bloomington, Indiana 47405 USA

**Keywords:** Psychology, Human behaviour

## Abstract

We analyze the visual perception task that home plate umpires (N = 121) perform calling balls and strikes (N = 3,001,019) in baseball games, focusing on the topics of perceptual learning and bias in decision-making. In the context of perceptual learning, our results show that monitoring, training, and feedback improve skill over time. In addition, we document two other aspects of umpires’ improvement that are revealing with respect to the nature of their perceptual expertise. First, we show that biases in umpires’ decision-making persist even as their overall accuracy improves. This suggests that bias and accuracy are orthogonal and that reduction of bias in decision-making requires interventions aimed specifically at this goal. Second, we measure a distinct difference in the rate of skill improvement between older and younger umpires. Younger umpires improve more quickly, suggesting that the decision task umpires engage in becomes routinized over time.

## Introduction

Over the course of more than 100 years, experimental psychologists have developed sophisticated methods for achieving laboratory control in the study of human judgment and behavior. However, this accumulated expertise may have had the unwelcome effect of predisposing them to neglect the possibilities of discovering principles of behavior by analyzing naturally occurring data sets rather than conducting experiments^1,2^. Uncovering principles of psychology by analyzing naturally occurring data is an exciting endeavor because (1) there has been a rise of well curated and large data sets involving collections of tagged images, text corpora, Wikipedia edit histories, trends in Twitter tag usage, demographics, consumer product sales, patent use and dependencies, sporting event outcomes, scientific citations, etc.^3^, (2) there now exist novel analytic methods for inferring causal relations from observational data^4^, (3) the data often come from strongly motivated decisions and life-changing behaviors of social importance^5^, and (4) the data sets allow us to explore the interplay between internal psychological processes and external environments, artifacts, and social institutions^6^.

Sporting contests offer particularly compelling naturally occurring data sets because of the expertise and life-long learning possessed by their participants and the highly incentivized behaviors that they capture^7^. Additionally, because of the widespread popularity of sports and the interest from both fans and professionals in objective assessments of performance, many sports leagues have made large investments in measurement and database technology that allow researchers to freely access copious amounts of well-curated data. For example, in the case of Major League Baseball (MLB) in the United States, the PITCHf/x monitoring system has been installed in every MLB baseball stadium since 2008, recording the trajectory of every pitch as it leaves a pitcher’s hand and the location of the pitch as it crosses the front of home plate. This detailed trajectory information is combined with the home plate umpire’s call, as well as contextual information regarding the score, previous calls, player information, time, date, inning, events, and pitch classification. Given the large number of individual pitches that a single umpire classifies as ball or strike during their MLB career, the dataset provides a highly diagnostic and robust source of information for revealing the mechanisms and biases underlying expert human perceptual judgment^8–10^. This is the dataset we use in this paper.

The perceptual judgment that we are interested in is made by the home plate umpire, positioned behind the catcher, in MLB baseball games. One of the jobs of this umpire is to determine whether the baseball as thrown by the pitcher passes through a volume of space called the “strike zone” when the batter does not swing at it. The left and right boundaries of this zone are defined by the left and right edges of the home plate, respectively. The top of the strike zone is defined by the midpoint between the top of the batter’s shoulders and the top of the batter’s pants. The bottom of the strike zone is defined by the hollow beneath the batter’s knee caps. Generally speaking, an umpire’s “strike” call (within the strike zone) benefits the pitcher’s team because the batter is called out after accumulating three strikes, and calling a pitch a “ball” (outside the strike zone) benefits the batter’s team because after accumulating four balls, batters advance to first base. A diagram depicting this perceptual task can be found in Fig. S1. Note that in the rulebook and in the diagram, the strike zone is a three-dimensional volume of space. In our models of the judgment task, we treat it as if it were merely a two-dimensional plane. This is a common simplification in studies of umpires and in casual discussion of baseball.

The detailed online archive of every MLB play in a season is a felicitous source of evidence bearing on these difficult perceptual judgments by these elite professionals. Given that the average speed of a MLB pitcher’s fastball is 91 to 94 MPH, a baseball takes only 450 milliseconds (ms) to reach the home plate, and is only above the home plate for about 10 ms. Accurate judgments by umpires about whether a pitch falls within a batter’s strike zone require years of deliberate practice^11,12^, strategic eye gaze patterns^13^, and complex coordination between perception, action, and judgment processes^14^.

It is not unusual for a professional MLB umpire to make more than 5,000 judgments in a single season, with some umpire careers spanning more than 40 years and 200,000 individual ball-strike decisions at the MLB level. Although umpires are generally accurate at this demanding perceptual classification task, with an average accuracy of 85% to 90% as measured by the PITCHf/x system, this accuracy varies depending on various factors and biases. For example, umpires tend to expand the strike zone for high-status, star pitchers relative to lower-status pitchers^15^, and shrink the strike zone for batters with more physical and social contact with umpires^8^. There is additional evidence that umpires expand the strike zone for pitchers playing in their team’s home stadium (home pitchers)^8^. Past work has also found that umpires’ decisions are sensitive to the preceding sequence of pitches^16^. When the count of strikes and balls on a batter favors the pitcher, umpires also shrink the strike zone for the subsequent pitch compared to when the count favors the batter^17^, consistent with the theory that umpires try to avoid making judgments that will have a large impact on the course of the game^8,18^. More recent work suggests that this behavior is consistent with maximizing accuracy^19^. Finally, while some evidence exists that umpires expand the strike zone for pitchers that match the umpire’s race^20^, other analyses do not show robust race-related biases^9,21^. All of these findings are based on the same dataset (recorded with the PITCHf/x system) that we use in our analysis.

Our primary contribution in this paper is to introduce a new model of umpires’ decision task, one that allows us to examine previously unmeasured features of it. Our model has two innovative features in particular. First, our psychophysical model features a parameter that measures umpires’ consistency in calling balls and strikes at the edge of the strike zone. Other measures of consistency have been presented in previous work, but none integrate it with other psychologically relevant features of the decision task^22^. Second, our model addresses deviations in the shape of the strike zone from the official rectilinear zone to one that allows vertical deviations to be compensated for by horizontal accuracy, and vice versa. Each of these phenomena, consistency and compensation, is represented by a unique parameter, allowing us to measure differences across umpires and over time.

Using the model, we make two substantive contributions to the psychological literature. Each contribution consists of empirical evidence, taken from observing these umpires, that provides useful input on important questions in psychology. First, we demonstrate that biases in umpires’ decision-making–specifically, tendencies to change the boundaries of their strike zones in different situations–persist even as their overall accuracy in calling balls and strikes improves. This supports the idea suggested in past literature that bias and accuracy are separable aspects of decision-making^23^. The umpires in our dataset received incentive-based training to become more accurate. While these incentives did improve accuracy, they left these biases unchanged. This demonstrates that if the goal of training is to decrease bias, then providing incentives for overall accuracy improvement is not always effective. Our second contribution is that we measure a distinct difference in the rate at which older and younger umpires improve in response to these incentives. Older umpires improve more slowly. This result corroborates findings in other application areas and documents age differences in novel aspects of psychophysical performance^24^. Specifically, we show this age difference in the consistency parameter described above.

## Methods

### Data

Pitch-tracking equipment, consisting of a system of cameras and known as PITCHf/x, was installed in every MLB stadium as of 2008. The data recorded by this system have been made publicly available for every pitch thrown since 2008 by MLB. We acquired this dataset from MLB’s data website, called Baseball Savant^25^. From this dataset, we are interested in the following variables for their relevance to the umpire’s perceptual task: the pitch location in the vertical plane at the moment it crosses home plate, measured in feet, with the ground and the horizontal center of the plate as the origin; and pitch metadata, including the count, the batter’s and pitcher’s handedness, the inning side, and which umpire made the call.

We restrict our analysis to 2008-2015. MLB installed new, radar-based tracking systems in 2016, and this change is associated with minor systematic changes in measurement accuracy and consistency^26^. We also restrict the data to pitches on which the batter did not swing, as these are the only pitches for which the umpire’s perceptual judgment is observed. The resulting dataset includes 3,001,019 pitches called by 121 umpires.

### Model

Previous models of umpires’ calls in MLB games have documented systematic shifts in called strike zones as a function of various contextual factors^8,16,18,20^. However, these models have not been based on a full psychophysical model of the perceptual judgment task confronting the umpire. This prior work used non-parametric or machine learning methods to analyze the strike zone and visualize its shape^8,19,27^. Those approaches achieve high predictive accuracy, but they do not measure meaningful parametric characteristics of the physical shape of the strike zone. A more recent empirical approach^28^ focuses more upon the shape of the zone, but does not address the psychophysical parameterization of decision making. Because we are interested in making inferences about the strike zone’s physical shape, we therefore construct a parametric model that extracts features of psychophysical interest based on a logistic function^29^ as:

1 $$\begin{aligned} P(\text {``strike''})=\text {logit}^{-1} \left( - \beta \left( d -\alpha \right) \right) \end{aligned}$$

where $P(\text {``strike''})$ is the probability of an umpire calling a pitch a “strike” instead of a “ball”; $\beta$ is the steepness of the transition from calling strikes to calling balls as the distance, $d$, of the pitch from the center of the strike zone increases; and $\alpha$ is the point of indifference, the distance at which the umpire is equally likely to call a ball or a strike. The point of indifference, $\alpha$, is defined in terms of our distance metric, not distance in Euclidean space. We define the distance $d$ as

2 $$\begin{aligned} d=\root r \of { \left| \left( x-x_{0} \right) \right| ^{r}+{\left| \left( \frac{y-y_{0}}{\lambda } \right) \right| ^{r}}} \end{aligned}$$

where $x$ and $y$ are the horizontal and vertical coordinates of the pitch, measured in feet, with the origin set at ground-level and the center of home plate. (These measurements are from the umpire’s perspective, so pitches with negative $x$ values will be closer to a right-handed batter than to a left-handed batter.) This definition of distance is useful because it allows for the shape of the strike zone to be flexible within a reasonable range. In particular, it allows the border of the strike zone to be a superellipse (a generalization of ellipses that, said roughly, allows for more squared-off corners) with variable height, width, eccentricity, and center. This modeling approach–restricting the strike zone to be superelliptical–is similar to that of Zimmerman et al.^28^ (Note also that we do not have measurements of the position of the umpire’s eyes when making the call, though this factor is relevant to their judgment. Fortunately, each umpire is in roughly the same position–with their head just behind the catcher’s helmet–for every call, so this should not substantially affect our analyses.) We estimate the model using the Bayesian statistical software Stan (Stan Development Team 2018).

### Interpretation of parameters

The parameters in this model represent meaningful measurements in the context of psychophysics and facilitate comparisons to the rulebook strike zone. One characteristic, **centering**, refers to how close the center of the umpire’s strike zone, point {$x_{0}$,$y_{0}$}, is to the rule-book center. A second characteristic, **strike zone size**, is directly quantifiable by the combination of $\alpha$ and $\lambda$. (Note that the parameter $\alpha$ represents the half-width, as $\alpha \lambda$ represents the half-height of the strike zone. In the results, we will present estimates of the full height and width, which are equal to $2\alpha$ and $2\alpha \lambda$, respectively.)

The parameter $\beta$ can be interpreted in terms of a third characteristic, **consistency**. A relatively large value of $\beta$ indicates two kinds of consistency. The first is that pitches that are further from the strike zone are not often called a “strike” when closer pitches are called “ball”. The second is that pitches that are thrown the same distance from the center of the strike zone tend to receive the same call. In the case of $\beta =\infty$, there is a single, sharp threshold distance such that a pitch is called a strike if and only if it is closer than this distance to the strike zone center. This parameter can also be interpreted as visual perceptual skill: it represents the accuracy with which umpires see the true location of the pitch.

A fourth characteristic, **rectilinearity**, is directly captured by $r$. The rule-book strike zone is completely rectilinear (corresponding to $r=\infty$), but if an umpire adopts a compensatory decision policy, in which a pitch being far from the horizontal center of the strike zone can be compensated for by it being very close to the vertical center, then their called strike zone might be better accommodated by a value of $2<r<\infty$. If umpires have difficulty separately appraising the horizontal and vertical deviations from the strike zone center, treating them as integrated together into an overall sense of distance from the strike zone center^30^, then a value of $r$ close to 2 would be expected^31,32^. Accordingly, a relatively high value of $r$ indicates that an umpire is successful at separately assessing the horizontal and vertical borders of the strike zone rather than reverting to a sense of overall distance that is imperfectly separated into horizontal and vertical components.

### Contextual covariates

We add contextual covariates to our model to control for various factors known to influence umpire calls. For each of the six parameters described above, we create a regression equation that includes fifteen independent variables in addition to an intercept: eleven indicator variables for the count (with zero to two strikes and zero to three balls possible, there are twelve unique counts, which necessitate eleven indicator variable in addition to the intercept), three indicator variables for the batter/pitcher handedness, and one indicator for the side of the inning (which indicates whether the home or away team is pitching). Because we are interested in the strike zones called by individual umpires, and the data are clustered at the umpire level, we also include a random intercept for each umpire.

Each umpire has six individual parameters, one for each of the six parameters described above ($\alpha$, $\beta$, $\lambda$, $r$, $x_{0}$, and $y_{0}$). We model these hierarchically, as samples from a multivariate normal distribution. The multivariate prior allows information to be shared between parameters: if umpires with large strike zone widths also tend to have large strike zone heights, then the multivariate structure will estimate that relationship. We discuss the covariates and the prior specification in more detail in the supplementary materials.

## Results

We present two primary findings in this paper. The first is that training did not reduce umpires’ perceptual biases even though it improved their perceptual skill. The second is that younger umpires displayed a faster rate of improvement in response to training. Before detailing those results, we provide a short overview of the model’s measurements pertaining to global changes in the strike zone. These measurements are consistent with the existing literature^8,33^, which suggests that our model provides an adequate fit to the data and, in our view, adds credence to those of our findings that are novel.

### Strike zone changes

**Figure 1:**
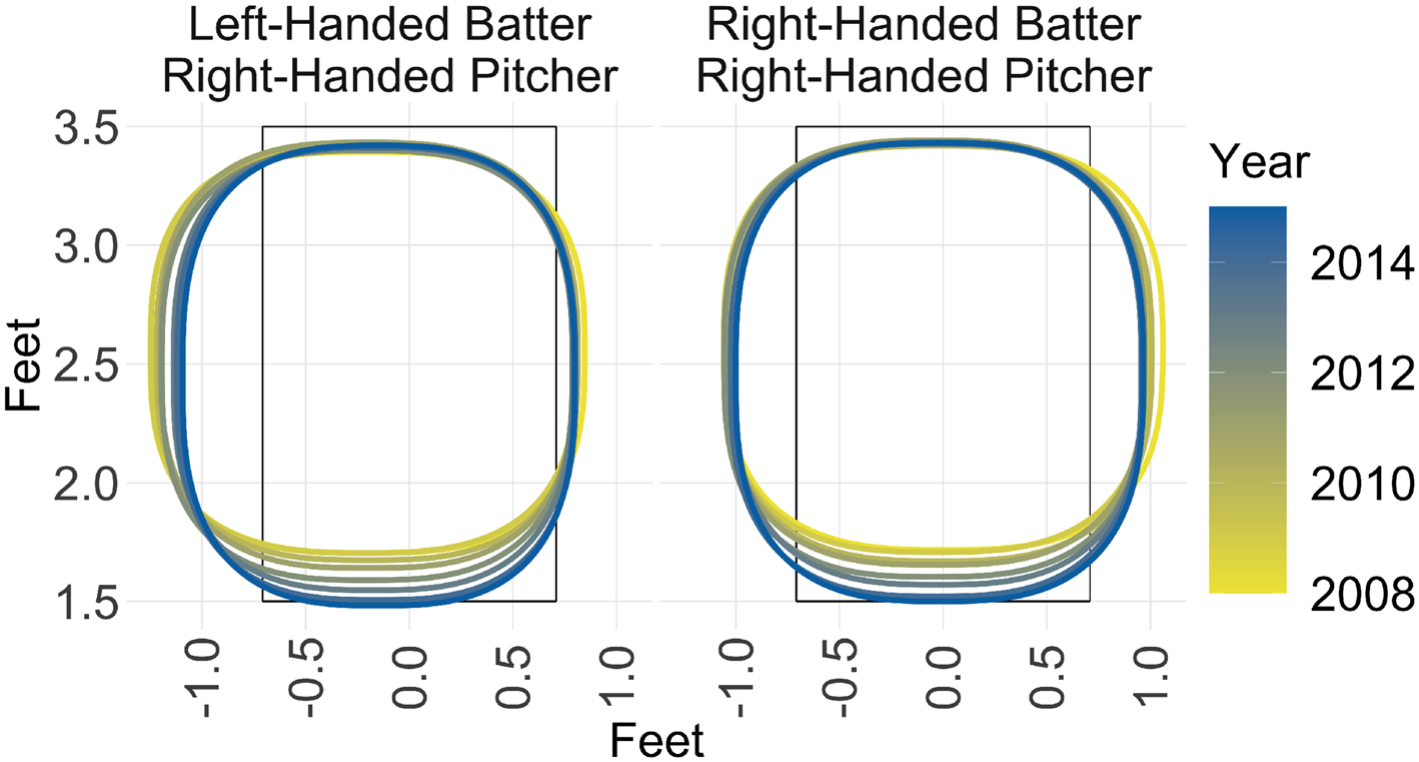
Time trends in strike zone shape (umpire’s point of view). The figure plots the 50% contour line (the point at which an umpire makes a strike call with probability 0.5) in a 0-0 count and in the top of the inning for each year from 2008 to 2015. The left-hand plot shows a left-handed batter’s strike zone and the right-hand plot shows a right-handed batters strike zone. The black boxes show the true strike zone. (Note that the top and bottom of the strike zone vary depending on the batter’s body position, so the top and bottom of the true strike zone in this diagram are just one realization of a possible true strike zone).

Consistent with past work^33^, from 2008 to 2015, MLB umpires made improvements in their ability to call the rulebook strike zone. Figure 1 shows a summary of the changes. The most salient change is that the bottom of the strike zone moved downward. The top of the strike zone was roughly correct in 2008 and remained stable, while the outside portion of the zone receded during this period. (Note that this figure shows a single contour line for each year, so it does not display changes in the consistency parameter, $\beta$. Changes in consistency would affect the distance between adjacent contour lines within a given year, indicating changes in the slope of the function that maps pitch location to the probability of a strike.)

**Figure 2:**
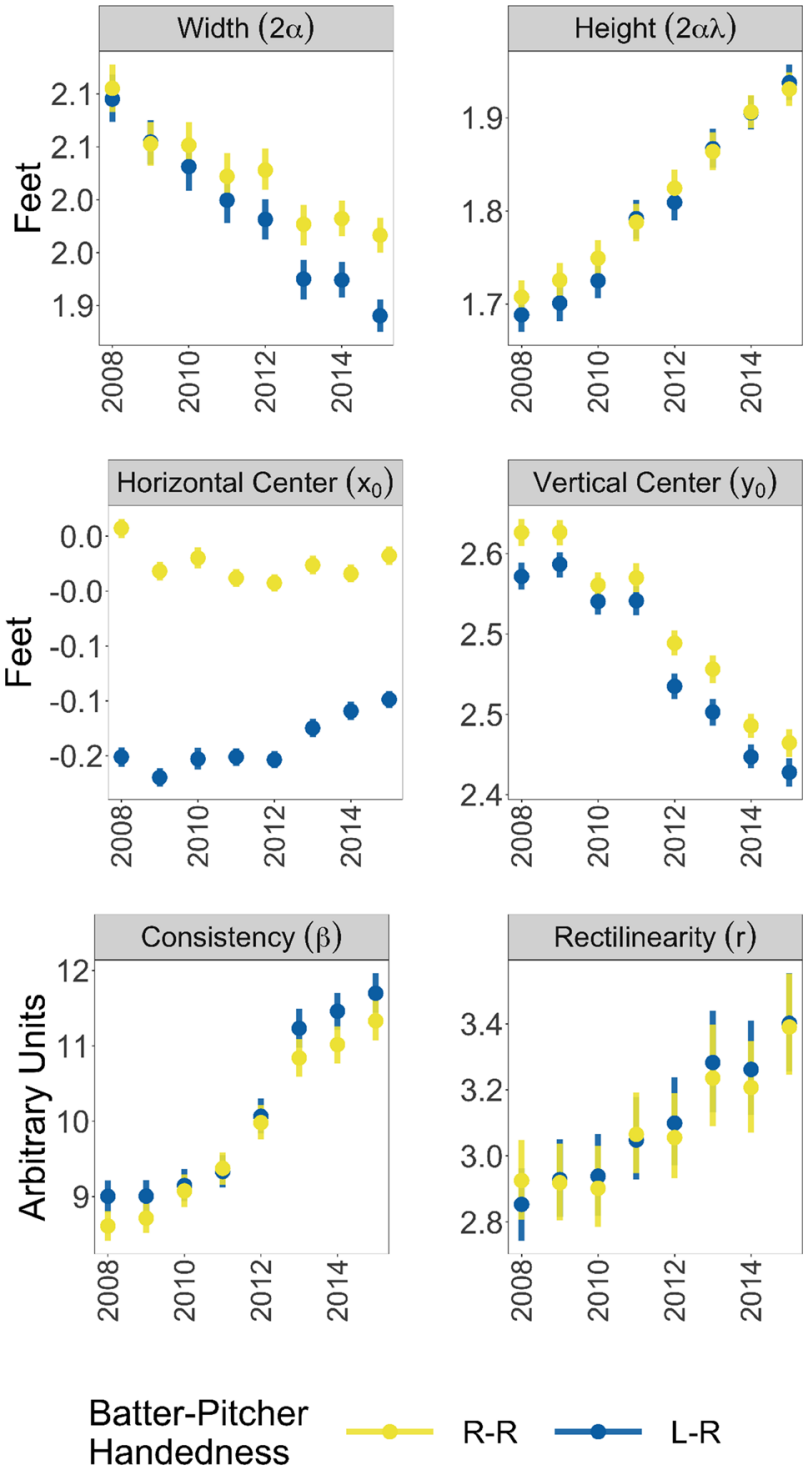
Time trends in parameter values. The width, height, and horizontal/vertical center parameters are measured in feet. The consistency and rectilinearity parameters are scaling factors and are measured in arbitrary units. The width and height measurements represent the full width and height of the strike zone, from one side to the other. (In the terminology of our model, this is twice the width and height parameters that enter the model, or $2\alpha$ for the full width and $2\alpha \lambda$ for the full height.) The horizontal/vertical center parameters are measured in reference to the origin, which is defined as the middle of home plate at ground level. The vertical bars in the plot represent 95% credible intervals.

Figure 2 shows the average parameter values in the generic context of a 0-0 count in the bottom of the inning (home team batting). For simplicity, we show the parameter estimates for a right-handed pitcher only, facing either a right-handed batter or a left-handed batter. Changing the pitcher’s handedness has only a small effect on each of the parameter estimates.

There is a consistent and substantial change in all of the parameter values (except for $x_0$, in plate appearances with right-handed batters). In all cases, the parameter estimates move toward the values stipulated by the rulebook strike zone. For example, the 2008 total strike zone width is about 2 feet, and by 2015 it is about 1.9 feet, a change of 1.2 inches towards the rulebook value of 1.42 feet. At the same time, the consistency parameter $\beta$ increased. This means that as the strike zone boundary moved closer to the rulebook values, umpires also became better at consistently calling that boundary.

### Lack of bias attenuation

Bias occurs when an umpire’s strike zone differs between different contexts. For example, an umpire who calls a wider strike zone when the home team is pitching–holding all other factors constant–is said to biased in favor of the home team. We define bias as the marginal effect of a covariate on the physical strike zone parameters in a given game situation (this definition is explained in detail in the supplementary materials).

**Figure 3:**
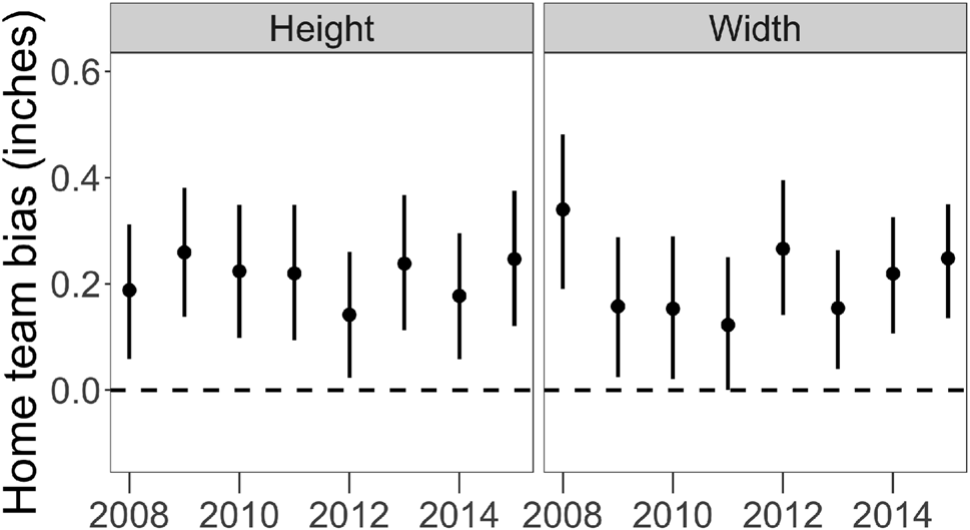
Measures of home-team bias over time. The y-axis shows the difference in inches between the height and width for a home-team pitcher, versus those for an away-team pitcher. Positive values indicate an advantage for the home team. The vertical bars show the 95% credible intervals.

Figure 3 shows the measurements of home-team bias in the height and width parameters from 2008 to 2015. These marginal effect estimates are calculated for the game situation of a 0–0 count with a right-handed pitcher and left-handed batter. (The main result we discuss here holds for other combinations of those parameters as well.) Figure 3 suggests that umpires are biased in favor of the home-team pitcher in both height and width, consistent with previous work^8,34^. We find no evidence of a consistent home-team bias in any of the other four strike zone parameters.

Central to our inquiry, we find that the magnitude of the bias is roughly constant over time, even as the model parameters overall are getting closer to the rulebook values. That is, even as umpires improve in their perceptual skill, the magnitude of their bias remains roughly constant. This suggests that despite the success of monitoring and training on accuracy and consistency, these interventions did not necessarily reduce bias. While we do not investigate racial bias, the result in Fig. 3 is in contrast to prior work arguing that umpires’ racial biases disappear when their performance is being monitored^20^.

We calculate analogous bias measurements for different counts, shown in Supplementary Fig. 1. As with home-team bias, our results are consistent with past work^8,15^ in that a significant bias does exist for several game contexts. The attenuation of bias is more complicated for count-based bias: some situational biases (e.g. the bias in strike zone width) are moderately reduced over time, while others (e.g. the bias in strike zone height) are essentially unchanged over time. This suggests that the relationship between skill improvement and bias attenuation is complicated. At a minimum, we can say that improvements in skill do not guarantee reductions in bias.

### Age and learning

Our goal in this section is to estimate the difference in learning rates between older umpires and younger umpires. To do so, we present a hierarchical Bayesian growth curve model that measures the difference in umpires’ rates of improvement based on their ages. We conclude that there is an association between age and learning, but that it is unclear what the mechanism is, for reasons to be discussed below. This builds upon prior work that found the same result using umpires’ global accuracy rates^33^. Our analysis extends that result by using the more-parsimonious psychophysical measurements of our strike zone model.

To motivate our analysis, we provide a short summary of the recent history of umpire training. A more detailed account can be found in Mills’ prior work^33^. Before umpires debut in the major leagues, they spend several years working in the minor leagues, which constitute professional baseball’s lower divisions. In 2008, MLB installed the PITCHf/x pitch-tracking system in all major-league stadiums (and not in minor league stadiums). (This is the system with which our data were measured.) In 2009, MLB and the umpires’ union agreed to a collective bargaining agreement that included performance incentives based on the PITCHf/x measurements, with the intention of encouraging umpires to adhere to the rulebook strike zone. This was the first substantial monitoring and training program that MLB instituted. Lastly, because the PITCHf/x system was only installed in major-league stadiums, umpires working in the minor leagues did not receive direct feedback based on a pitch-tracking system until they reached the major leagues (or, in some cases, when working MLB Spring Training games).

Given this history, in our analysis we only consider umpires who debuted during or before 2008. Other umpires, even though they did not receive direct feedback from the PITCHf/x system prior to their respective MLB debuts, may have been indirectly influenced by the new, PITCHf/x-based observations of MLB umpires’ performance. That is, there may have been a trickle-down effect in which minor league umpires received instructions based on the new observations of major league umpires. If this is the case, then umpires who debuted after 2008 are not exchangeable with umpires who debuted during or prior to 2008. Their rates of improvement in response to the PITCHf/x-based training system could be affected by their training experience at the minor league level. In the supplementary materials, we provide a robustness check that shows that our substantive result does not depend on this modeling decision.

A second advantage of this restriction is that the resulting set of umpires begins observation at the same time–2008, when PITCHf/x was first installed. If we were to include later-debuting umpires, their observation periods would begin later than 2008. Umpires who debuted after 2008 may have been indirectly affected by the PITCHf/x-based training program prior to our observation of them, and so we remove them from this analysis. These umpires would have worked in the minor leagues between 2008 and their individual debut years. Though they did not receive direct strike zone feedback during that time, they may have received general directives about how to adjust their strike zones based on the evidence from the major-league level. (For example, MLB may have directed all umpires to raise the strike zone based on observations of MLB umpires.) There is evidence that umpires change their behavior based on such general directives, even in the absence of feedback: in 2001, MLB told umpires to call more strikes and the strike rate suddenly increased (see Mills’ prior work^33^, sec. 3). If indirect feedback does affect performance and those umpires received such feedback, then including those umpires in the age analysis would bias our estimate of the age and learning association, because they are systematically different from umpires who did not receive that indirect feedback. Out of 121 total umpires in our dataset, 35 umpires debuted after 2008, leaving us with 86 umpires in this analysis. The unit of observation in our analysis is an umpire-year, and we retain 641 umpire-years out of 773 in the full dataset. Among these umpires, in 2008 the average umpire was 44 years old, the youngest was 30, and the oldest was 62.

Moving to the growth curve model, the dependent variable is each umpire’s $\beta$ parameter in each year. Out of the six strike zone parameters, we chose $\beta$ because it represents the most general measurement of umpires’ performance. For each umpire, we assume that, once the PITCHf/x-based training begins, his $\beta$ parameter changes linearly as a function of time. The linear rate-of-change in each umpire’s $\beta$ parameter constitutes his learning rate. We estimate these rates-of-change using a hierarchical Bayesian model with umpire-specific parameters, following the framework laid out in^35^. (We use masculine pronouns because all umpires in the dataset are men. Women have umpired MLB games in spring training at various times, but never in the regular season).

In the dataset for our cohort analysis, the unit of observation is an umpire-year. For each umpire in each year, we produce an estimate of the umpire’s $\beta$ parameter in a 0-0 count, with a right-handed batter and a right-handed pitcher, in the top of an inning. We call this quantity $\beta ^\dagger _{ut} = \exp \left( \beta _{0-0,t} + \beta _{\text {RHB-RHP,t}} + \beta _{\text {TOP,t}} + \beta _{ut}\right)$, for each umpire $u$ and each year $t$. (This simulated quantity is calculated using the mean value of the joint posterior distribution of the parameters. Technically, this exaggerates the certainty in our estimates because we collapse each umpire’s posterior distribution to a point estimate. However, given that our estimates of the umpires’ consistencies are relatively precise and because it simplifies the cohort model relative to one that fully incorporates the uncertainty, we think this is a reasonable approximation.) This value, $\beta ^\dagger _{ut}$, is the dependent variable. The dataset also includes each umpire’s year of birth and the year of his MLB debut.

Our model is a two-level hierarchical model. At the first level, for which the observational unit is umpire-years, the dependent variable is the quantity $\beta ^\dagger _{ut}$. The first level has an umpire-specific intercept term $\theta _{0u}$ and one independent variable, the timepoint $t$. For all umpires, $t=0$ is the year 2008 and $t$ increases by one for each additional year the umpire appears in the dataset. The umpire-specific slope parameter $\theta _{1u}$ therefore measures the annual rate-of-change in the $\beta ^\dagger$ values for umpire $u$. The parameter $\theta _{1u}$ estimates the learning rate for umpire $u$, and it is the outcome we are interested in: is this value associated with age?

At the second level, the individual growth curve parameters, $\theta _{0u}$ and $\theta _{1u}$, are given a multivariate normal prior with mean dependent on the umpire’s (mean-centered) birth year. The $\gamma$ parameters are given normal priors with mean 0 and a standard deviation of 100 (reflecting low prior certainty). The covariance matrix $\mathbf {\sigma _\theta }$ is given a Lewandowski, Kurowicka, and Joe (LKJ) prior with parameter 2^36^. We give the parameter $\sigma _y$ a Cauchy prior with location parameter 0 and scale parameter 5.

The $\gamma$ parameters have straightforward interpretations in terms of an individual umpire’s development. The parameter $\gamma _{00}$ is the $\beta ^\dagger$ value in year $t=0$ for an umpire with the average umpire birth year. The intercept parameter $\gamma _{10}$ measures the average rate-of-change of all the umpires. The slope parameter $\gamma _{01}$ measures the relationship between umpire birth year and umpire consistency in 2008, the first year of observation. Lastly, $\gamma _{1,1}$ measures the relationship between umpire birth year and the rate-of-change in umpire consistency. The slope parameter $\gamma _{1,1}$ is the key parameter in our age analysis. If this parameter is non-zero, then there is a difference in the learning rate between old and young umpires.

The key elements of the model are shown below. The variable $B_u$ represents the mean-centered birth year of umpire $u$.

$$\begin{aligned}{} & {} \beta ^{\dagger }_{ut} \sim N\left( \theta _{0u} + \theta _{1u}t, \sigma _y \right) \\{} & {} \left[ \begin{array} {r} \theta _{0u} \\ \theta _{1u} \end{array}\right] \sim N\left( \left[ \begin{array} {r} \mu _{0u} \\ \mu _{1u} \end{array} \right] , \mathbf {\sigma }_\theta \right) \\{} & {} \mu _{0u} = \gamma _{00} + \gamma _{01} \textit{B}_u\\{} & {} \mu _{1u} = \gamma _{10} + \gamma _{11} \textit{B}_u \end{aligned}$$

**Figure 4:**
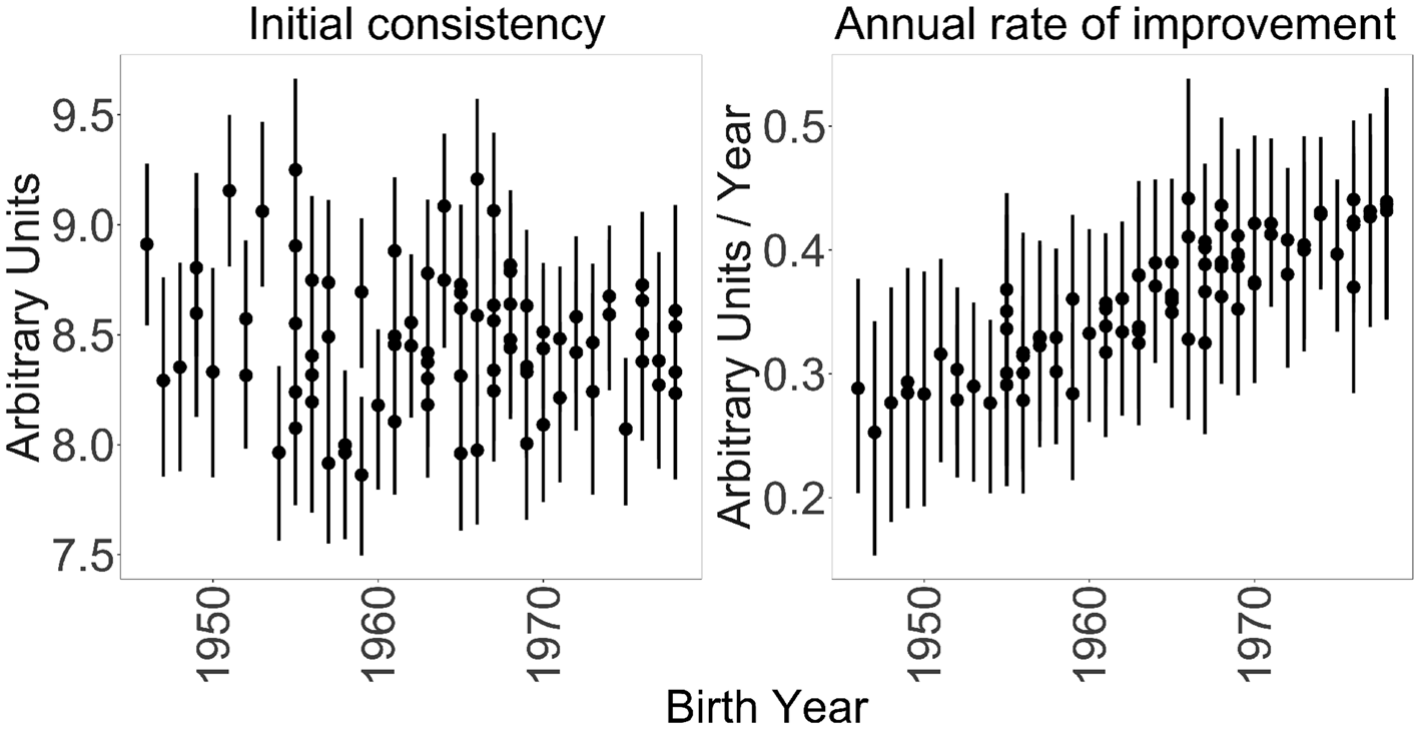
Cohort effect model estimates. The vertical bars show the 95% credible intervals for each individual umpire’s parameter estimates. The left plot shows each umpire’s consistency measurement in 2008, the first year of PITCHf/x-based observation. The right plot shows each umpire’s rate of improvement in consistency over the years during which he was observed by the PITCHf/x system.

Figure 4 shows the estimates of $\theta _{0u}$ and $\theta _{1u}$. The upward slope in the right-hand panel indicates that there is a relationship between age and learning rate. As described above, the parameter $\gamma _{11}$ measures that relationship. Its posterior median is 0.006 and the 95% credible interval is (0.003,0.008), indicating a high degree of confidence that there is a positive association between birth year and learning rate. That is, the younger an umpire is (the higher his birth year), the faster his $\beta$ parameter improves during training. There is not evidence for an association between umpires’ birth years and their initial consistency, suggesting that the old and young umpires are comparable prior to the start of training: the posterior median for $\gamma _{01}$ is -0.005 and its 95% credible interval is (−0.019,0.011).

The magnitude of this difference is best illustrated through an example. Consider a 30-year old umpire and a 60-year umpire who, prior to training, have the same initial consistency of 8.5. Assuming that our estimate of 0.006 is the true value of $\gamma _{11}$, then after ten years of training, the 30-year old umpire will improve his consistency by 4 units (a 47% improvement over the initial value) and the 60-year old umpire will improve his consistency by 2.1 units (a 25% improvement over the initial value). The thirty-year age difference corresponds to almost double the magnitude of learning.

## Discussion

We present two main findings in this paper. First, improvements in perceptual accuracy, resulting from feedback on performance, do not guarantee bias attenuation. This result is strongest in the measurements of umpires’ bias in favor of home teams (Figure 3), but appears in the results on count bias as well (see Figure S1). From 2008 to 2015, the average strike zone width changed by about 2.5 inches, but during that time umpires’ bias in favor of home team pitchers remained constant at about 0.2 inches. For count bias, some instances of bias were reduced (e.g. in 3-2 counts the bias is completely attenuated by 2015), but most remain constant. Furthermore, the lack of bias attenuation occurred even as umpires increased their consistency by roughly 33%. Given that umpires are “seeing” the ball more accurately over time, it is surprising that their home-team bias remains constant.

The persistence of bias demonstrates that accuracy and bias are separable and orthogonal features of decision making^23^. Improvements in overall accuracy do not guarantee improvements in bias. This has important implications for efforts to adapt decision-makers’ behavior. For example, if MLB wanted to reduce these context-based biases, then they would need to give umpires incentives specifically for that purpose instead of (or in addition to) incentives for overall accuracy.

The persistence of these biases, coincident with improvements in perceptual accuracy, suggests that the biases are manifest at a post-perceptual decisional stage. If the biases were directly influencing pitch perception, then their magnitude would probably be expected to decrease as perceptual judgments became increasingly precise over the years. Instead, the biases are likely affecting decisional thresholds for calling pitches “strikes” vs “balls” rather than distorting the perceived locations of pitches.

Second, these results add to the literature on the effects of aging in occupational training^24^. In particular, there is often a loss of perceptual processing speed with increasing adult age^37^. Meta-analyses have shown that, in general, training proceeds more efficiently for younger than older adults in occupational settings^38^. A training perspective is particularly relevant for MLB home plate umpires because a major professional change was enacted in 2008 in the form of the PITCHf/x pitch measurement technology that was widely distributed to ballparks across the USA. Their introduction led to a sudden increase in precise feedback given to umpires, leading to rapid increases in the consistency with which umpires called pitches, as measured here by $\beta$. Consistent with the literature on systematic reductions in learning efficiency and perceptual skills with age^39–41^, consistency improved significantly more rapidly for younger than older umpires. Our results confer evidence against this aging difference being caused by different initial levels of performance, as there were no differences in umpires’ initial consistency as a function of age. The more rapid improvement in consistency for younger umpires may be because of their greater fluid intelligence resources^40,42^, the greater need for older umpires to unlearn previously learned cognitive and perceptual routines^43^, or a combination of these age and experience factors. Our study cannot distinguish between these underlying causes and this is not an exhaustive list of potential explanations. Further work is needed to determine the reasons for the difference. Although training advantages for younger compared to older adults are often greater in laboratory experiments than field research^38^, our analysis shows a robust, quantitative training effect for both younger and experienced professionals who have risen to the very top echelon of their professions.

### Supplementary Information

Supplementary Information.

## Data Availability

The datasets used in this study are available for download in the OSF repository at https://osf.io/hv68j/.
