# Supplementary material for: The psychophysics of home plate umpire calls
Source: Sci Rep. 2024 Feb 1;14:2735. doi: 10.1038/s41598-024-52402-y (PMC10834529; doi:10.1038/s41598-024-52402-y)
Supplement: Supplementary file 1 — Supplementary Information. [file 41598_2024_52402_MOESM1_ESM.pdf]

Supplementary Materials

Physical Model

Contextual Covariates

As described in the main text, each of the physical parameters is regressed on a vector of contextual covariates. These covariates are the count, the combination of batter and pitcher handedness, and the side of the inning. The covariates have, respectively, twelve levels, four levels, and two levels. We also include an umpire-specific intercept, to measure the tendencies of individual umpires. One extension of this model would be to interact the umpire-specific terms with the other covariates, as in model 5 of [2]. That would allow us to measure each umpire's strike zone within each context. However, the simpler model, without interaction terms, provides an adequate baseline for measuring differences between umpires, assuming that there are not large interactive differences between umpires. The regression equations are shown in Equation 1. As noted in the main text, β, α, λ, r, and y_0 are restricted to be positive. We make this restriction by regressing the log of the parameter on the covariates. These covariates measure changes in the strike zone between different contexts.

$$\begin{aligned}
\log\beta &= \beta_{\text{intercept}} + \beta_{\text{count}} + \beta_{\text{platoon}} + \beta_{\text{side}} + \beta_{\text{umpire}} \\
\log\alpha &= \alpha_{\text{intercept}} + \alpha_{\text{count}} + \alpha_{\text{platoon}} + \alpha_{\text{side}} + \alpha_{\text{umpire}} \\
\log\lambda &= \lambda_{\text{intercept}} + \lambda_{\text{count}} + \lambda_{\text{platoon}} + \lambda_{\text{side}} + \lambda_{\text{umpire}} \\
\log r &= r_{\text{intercept}} + r_{\text{count}} + r_{\text{platoon}} + r_{\text{side}} + r_{\text{umpire}} \\
x_0 &= x_0^{\text{intercept}} + x_0^{\text{count}} + x_0^{\text{platoon}} + x_0^{\text{side}} + x_0^{\text{umpire}} \\
\log y_0 &= y_0^{\text{intercept}} + y_0^{\text{count}} + y_0^{\text{platoon}} + y_0^{\text{side}} + y_0^{\text{umpire}}
\end{aligned} \tag{1}$$

Prior Distributions

To complete the Bayesian model, we apply prior distributions to the model parameters. We apply standard normal priors, $N(0,1)$, to each of the contextual covariates in Equation 1, excluding the global intercept terms and the umpire-specific intercept terms. The global intercept terms receive informative prior distributions as described below. The umpire-specific terms receive a hierarchical prior distribution as follows. For each umpire, we construct a 6-dimensional vector, called $\boldsymbol{\zeta}_u$, that contains the umpire's intercept for each of the six strike zone parameters. These $\boldsymbol{\zeta}_u$ vectors are drawn from a hierarchical multivariate normal prior with mean $\boldsymbol{\mu}_{\boldsymbol{\zeta}}$ and variance-covariance matrix $\boldsymbol{\Sigma}_{\boldsymbol{\zeta}}$, as in Equation 1. We give each element of the hyperparameter $\boldsymbol{\mu}_{\boldsymbol{\zeta}}$ a normal prior centered on 0 with variance 1 and we give $\boldsymbol{\Sigma}_{\boldsymbol{\zeta}}$ an LKJ prior with parameter value 2 [3].

$$\boldsymbol{\zeta}_u \sim \text{MvNormal}(\boldsymbol{\mu}_{\boldsymbol{\zeta}}, \boldsymbol{\Sigma}_{\boldsymbol{\zeta}})$$

Conveniently, the rule book strike zone provides specific physical measurements of the strike zone that constitute a good initial guess at the true strike zone parameters. Our prior distributions are based on the values set by the rulebook. The rulebook stipulates that the width of the strike zone is 17 inches. Because the α parameter measures one half the width of the strike zone, we expect it to be about $\frac{17}{24}$ feet. Based on this, we give the $\alpha_{\text{intercept}}$ parameter a normal prior centered on $\log\left(\frac{17}{24}\right) = -0.34$ with variance 1.

$$\alpha_{\text{intercept}} \sim N(-0.34, 1)$$

The height of the strike zone depends on the batter's stance, but it is generally about two feet high. In our model, the height of the strike zone is equal to $2\lambda\alpha$, which suggests a λ value of $\frac{24}{17}$. We give the $\lambda_{\text{intercept}}$ parameter a normal prior centered on $\log\left(\frac{24}{17}\right) = 0.34$ and variance 1.

$$\lambda_{\text{intercept}} \sim N(0.34, 1)$$

Based on the rulebook definition, the x_0 parameter should be 0. Like the λ parameter, the y_0 parameter will change depending on the stance of each batter. Generally, the vertical center of the strike zone will be at about 2.5 feet. We give the $x_0^{\text{intercept}}$ parameter a normal prior distribution centered on 0 with variance 1 and give the $y_0^{\text{intercept}}$ parameter a normal prior distribution centered on $\log 2.5 = 0.92$ with variance 1.

$$x_0^{\text{intercept}} \sim N(0, 1)$$
$$y_0^{\text{intercept}} \sim N(0.92, 1)$$

For the β and r parameters, the rulebook stipulates values of $-\infty$ and ∞, respectively. As these are parameters of special importance in the context of studying perception, we want to avoid influencing the outcome with our choice of prior. For that reason, we choose relatively diffuse priors. For the $\beta_{\text{intercept}}$ parameter we use a normal prior with mean 4 and variance 1. For the $r_{\text{intercept}}$ parameter we use a normal prior centered on 1 with variance 1. The priors on β and r are difficult to evaluate in substantive terms. Because the parameters are unit-free scaling factors, the substantive meaning of each parameter's scale is not straightforward. We think that our choices of priors are reasonable for at least two reasons. First, there are enough data to overwhelm the priors. Second, we are primarily interested in changes over time and we apply the same prior to each year. If we are introducing unreasonable bias through our priors, then we are introducing the same bias in each year.

$$\beta_{\text{intercept}} \sim N(4, 1)$$
$$r_{\text{intercept}} \sim N(1, 1)$$

Definition of Bias

For each strike zone parameter, bias is defined to be the difference in that parameter's value between two combinations of covariates–corresponding to two in-game contexts–that differ in only one of the covariates. For example, we estimate home-team bias by comparing each strike zone parameter value when the home team is pitching to the parameter value when the away team is pitching, while holding all other covariates constant. Each measure of bias must be defined for a specific strike zone parameter. We can estimate home-team bias in strike zone width, height, consistency, etc.

In the paper, we measure home team bias for the game context of a right-handed pitcher and right-handed batter, and in a 0-0 count. The home-team bias in strike zone width, which we call $\tau_{\text{Home},\alpha}$, is defined as

$$\tau_{\text{home},\alpha} = \exp(\alpha_{\text{intercept}} + \alpha_{\text{0-0}} + \alpha_{\text{RHB-RHP}} + \alpha_{\text{top}}) - \exp(\alpha_{\text{intercept}} + \alpha_{\text{0-0}} + \alpha_{\text{RHB-RHP}} + \alpha_{\text{bottom}})$$

Technically, $\alpha_{\text{0-0}} = \alpha_{\text{top}} = 0$ because a 0-0 count is the reference value for α_{count} and the top of the inning is the reference value for α_{side}. We include those terms in our explanation for completeness. This measure will be larger when umpires favor home-team pitchers, as the first term is the width parameter given to home-team pitchers and the second term is that given to away-team pitchers.

We calculate count bias for each count relative to a 0-0 count. For example, bias in width in a 3-0 count, called $\tau_{\text{3-0},\alpha}$, is defined as

$$\tau_{\text{3-0},\alpha} = \exp(\alpha_{\text{intercept}} + \alpha_{\text{3-0}} + \alpha_{\text{RHB-RHP}} + \alpha_{\text{top}}) - \exp(\alpha_{\text{intercept}} + \alpha_{\text{0-0}} + \alpha_{\text{RHB-RHP}} + \alpha_{\text{top}})$$

All measures of bias that we present are calculated in this way, other than those for x_0. Note that bias in x_0 can be measured directly as the difference in parameter estimates for different values of each covariate

because the exponential function does not appear in the calculation of x_0. For example, home-team bias in strike zone width is

$$\tau_{\text{home},x_0} = (x_0^{\text{intercept}} + x_0^{\text{0-0}} + x_0^{\text{RHB-RHP}} + x_0^{\text{top}}) - (x_0^{\text{intercept}} + x_0^{\text{0-0}} + x_0^{\text{RHB-RHP}} + x_0^{\text{bottom}}) = x_0^{\text{top}} - x_0^{\text{bottom}}$$

Which, in this case, is just $-x_0^{\text{bottom}}$. This is because $x_0^{\text{top}} = 0$, as it is the reference value for x_0^{side}.

Robustness Checks

In this section, we report two additional specifications of the cohort model to show that the substantive result does not depend on our decision to restrict the dataset to umpires who debuted in 2008 or earlier. Our first model is identical to the model presented in the main paper, but it includes the entire set of umpires. The results are shown in Figure S3. The primary substantive result, that older umpires improve more slowly, persists. This is apparent in the right-hand panel.

The primary difference between the results of this model and those of the model from the main paper is that umpires who debuted after 2008 have higher starting values of β. That is, when they debut, they have higher consistency than their older counterparts had in their own debuts. This is probably a combination of two factors: first, as we suggested in the main paper, training at the minor league level may depend indirectly on observations derived from the PITCHf/x system. That is, the umpires who debut after 2008 received better training while in the minor leagues than their older counterparts did. Second, for an umpire who debuted after 2008, the average consistency of an MLB umpire was higher at his debut than it was in 2008. This is a result of the general skill improvements we report in this paper. Presumably, this means that after 2008, minor league umpires had to display a higher skill-level on this particular skill in order to qualify for the major leagues, possibly with higher weight placed on these more objectively measured ball-strike calls relative to previous years.

Our second robustness check model includes the entire set of umpires but adds an additional covariate and an interaction term at the second level of the hierarchical model. The additional covariate is an indicator variable that is a one if the umpire debuted after 2008 and a zero if the umpire debuted during or before 2008. Given the point made in the last paragraph, that the threshold for entry into the major leagues may have changed after 2008, it is unreasonable to model post-2008 debutees' initial consistency measurements as exchangeable with pre-2008 debutees' measurements. Thus, we model them with an interaction term. The results of this model are shown in Figure S4. The new specification of the second-level regression is as follows. The variable D_u is the indicator variable for whether umpire u debuted after 2008 versus during or prior to 2008.

$$\begin{aligned}\mu_{0u} &= \gamma_{00} + \gamma_{01}B_u + \gamma_{02}D_u + \gamma_{03}B_u \times D_u \\ \mu_{1u} &= \gamma_{10} + \gamma_{11}B_u + \gamma_{12}D_u + \gamma_{13}B_u \times D_u\end{aligned}$$

Again, the main substantive result persists in this model. The primary difference between the results of this model and those of the preceding model is that the relationship between age and rate-of-improvement among umpires who debuted after 2008 changes. In the first model it was similar to that for all umpires (this is not surprising, given that the hierarchical assumption shrank the estimates for these umpires towards the linear relationship fit with all umpires). In this model, it appears to be negative, which would indicate that the youngest set of umpires is actually getting less consistent over time. We suspect that this is not actually the case and that this negative estimate is a random result. The uncertainty in the estimates for the post-2008 debuting umpires is large, which makes sense given that we have fewer years of observation for those umpires.

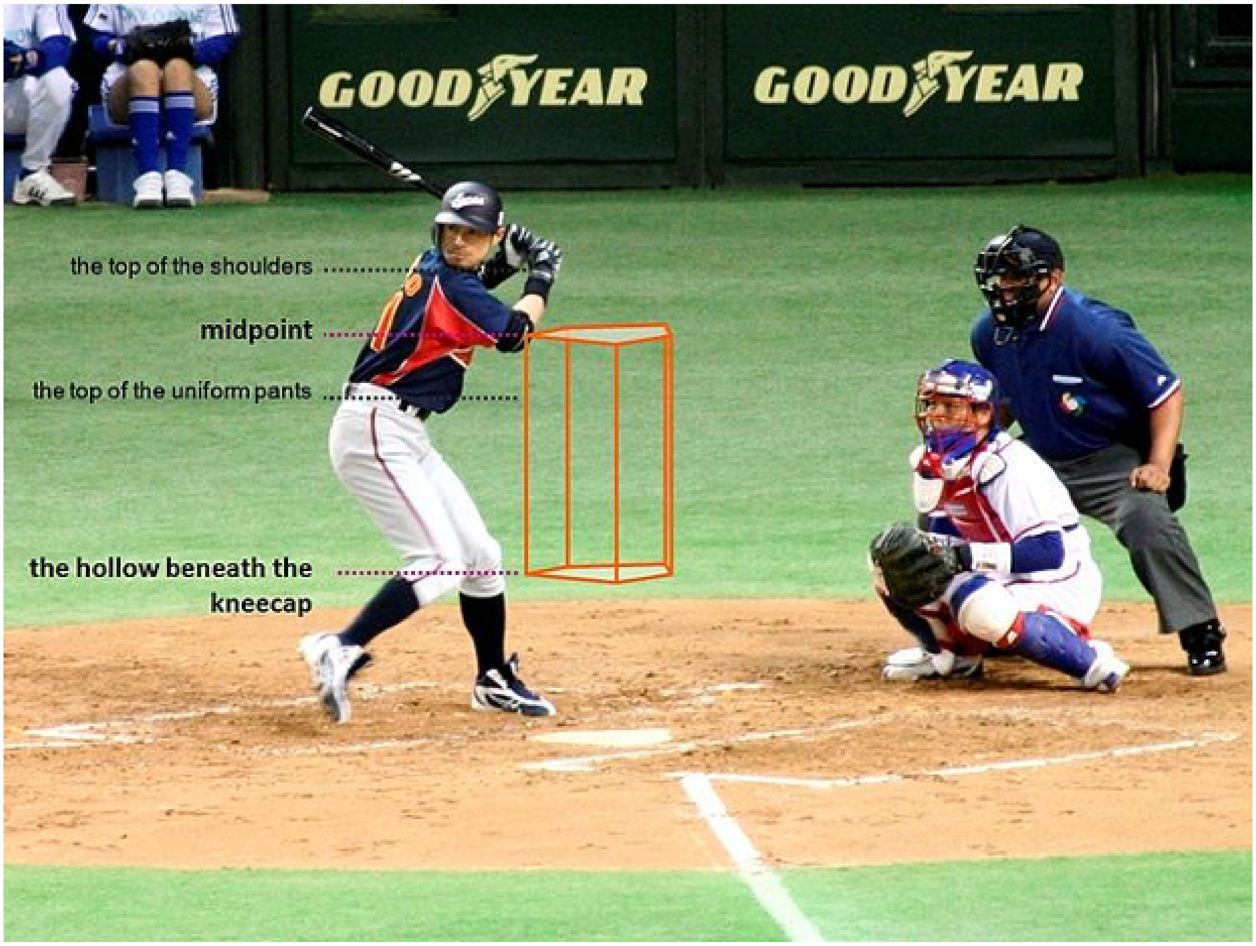

Figure S1: Illustration of the strike zone and the umpire's judgment task. The strike zone is a three-dimensional space floating above the edges of homeplate. According to the MLB rulebook, it's height is determined by the batter's body position, such that the bottom of the strike zone is at the hollow below the batter's knee and the top is at the midpoint between the batter's should and the top of the batter's pants. (This image is under a Creative Commons 2.0 license and is available online [1].)

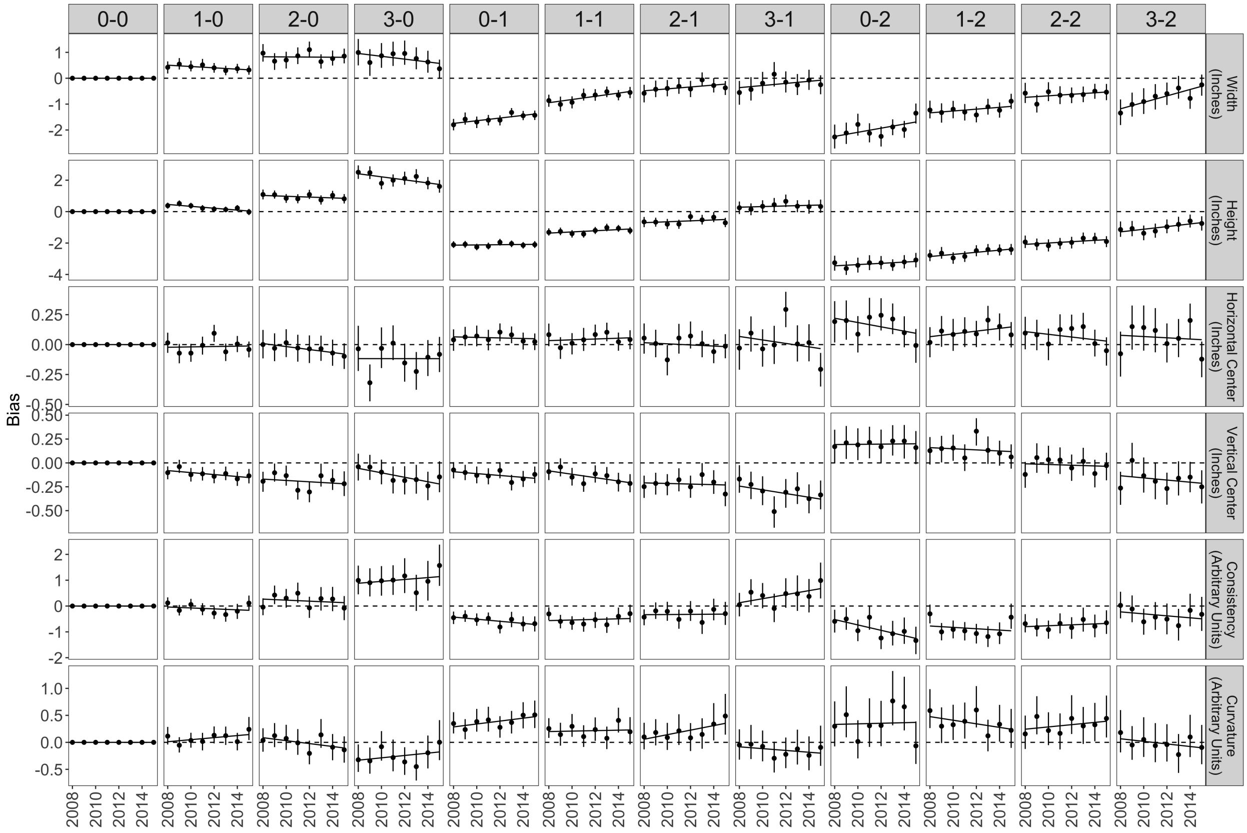

Figure S2: Count bias measurements. The y-axis shows the magnitude of bias, with different units for each row. The units are listed on the right-hand side, as subheadings below the parameter labels. Positive values indicate a higher value of the parameter for the count indicated by the column relative to a 0-0 count. (For this reason, the values in the 0-0 column are all zero.) Solid black lines show linear regression fits to the data within each cell. The dashed line in each cell indicates zero bias.

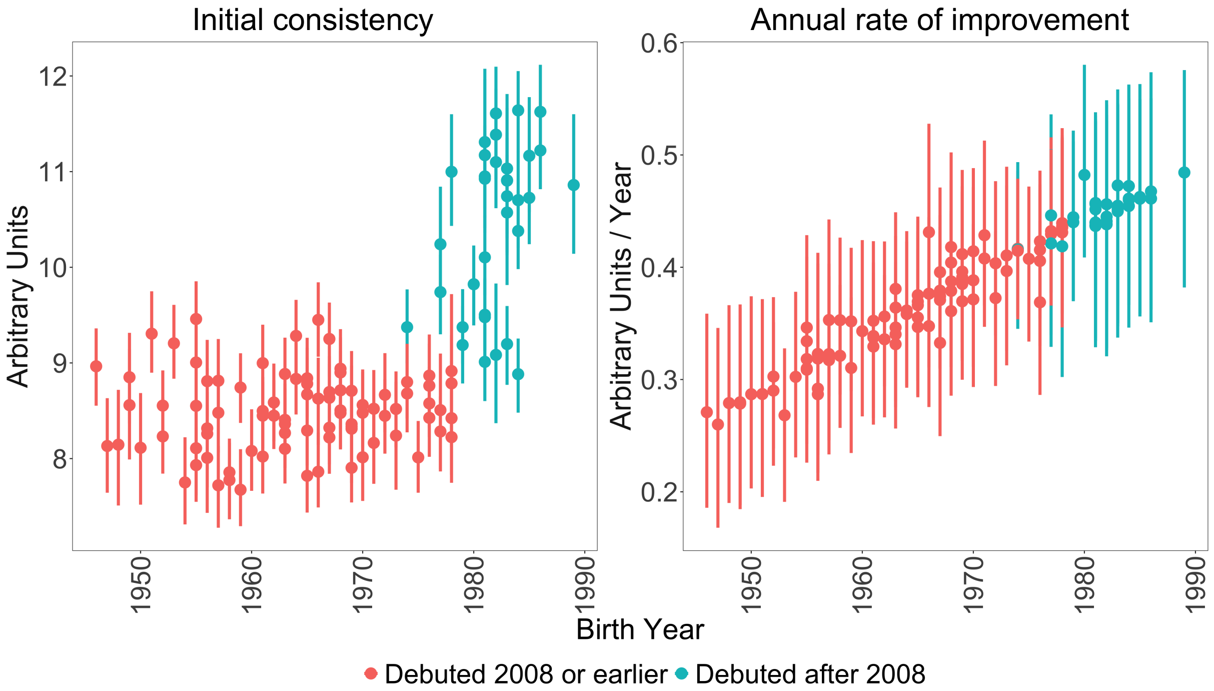

Figure S3: These are the results from the first cohort analysis robustness model. The left plot shows the relationship between birth year and an umpire's initial consistency in his first year of observation under the PITCHf/x system. The right hand plot shows the relationship between an umpire's birth year and his rate of improvement in consistency over the time during which he is observed by the PITCHf/x system.

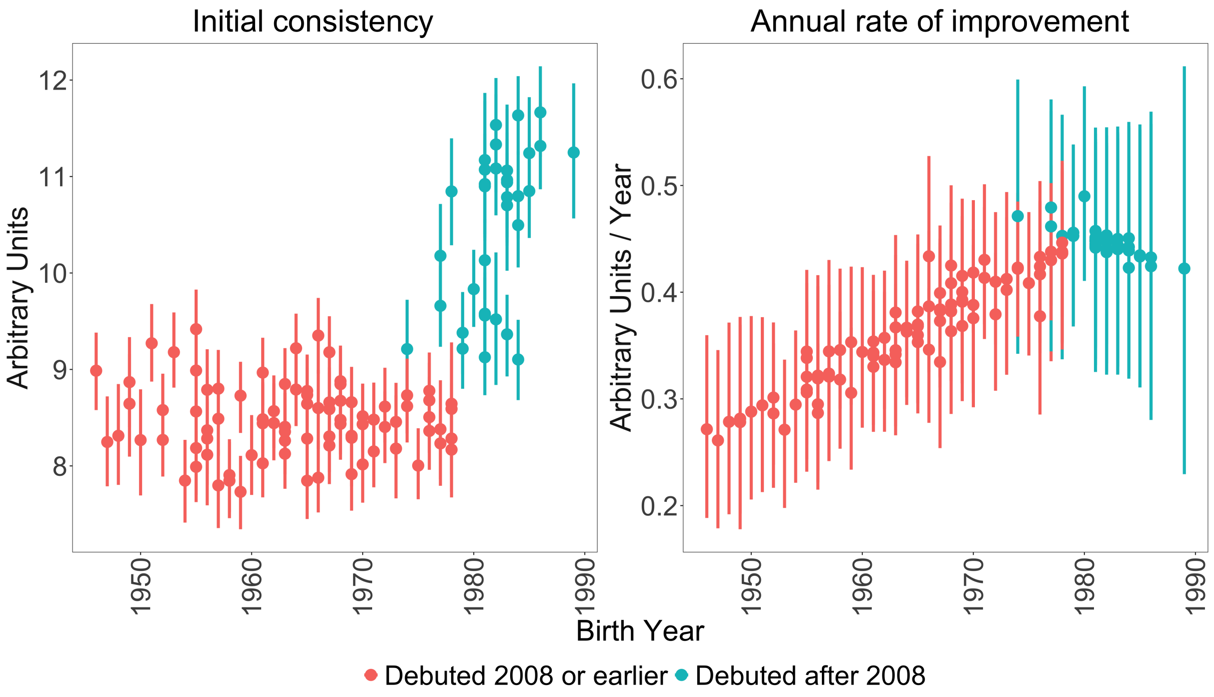

Figure S4: These are the results from the second cohort analysis robustness model. The plots are analogous to those in the preceding figure.

References

[1] Wikimedia Commons. *Strike zone*. 2008. URL: https://upload.wikimedia.org/wikipedia/commons/8/89/Strike_zone_en.JPG.

[2] Sameer K. Deshpande and Abraham J. Wyner. “A Hierarchical Bayesian Model of Pitch Framing”. In: *arXiv:1704.00823 [stat]* (Apr. 2017). arXiv: 1704.00823. URL: http://arxiv.org/abs/1704.00823.

[3] Daniel Lewandowski, Dorota Kurowicka, and Harry Joe. “Generating random correlation matrices based on vines and extended onion method”. In: *Journal of Multivariate Analysis* 100.9 (Oct. 2009), pp. 1989–2001. ISSN: 0047-259X. DOI: 10.1016/j.jmva.2009.04.008. URL: http://www.sciencedirect.com/science/article/pii/S0047259X09000876 (visited on 08/08/2019).
